# Supplementary material for: Green Synthesis of Silver Nanoparticles and Its Combination with Pyropia columbina (Rhodophyta) Extracts for a Cosmeceutical Application
Source: Nanomaterials (Basel). 2023 Mar 10;13(6):1010. doi: 10.3390/nano13061010 (PMC10054154; doi:10.3390/nano13061010)
Supplement: Supplementary file 1 [file nanomaterials-13-01010-s001.zip › nanomaterials-2243560-supplementary.pdf]

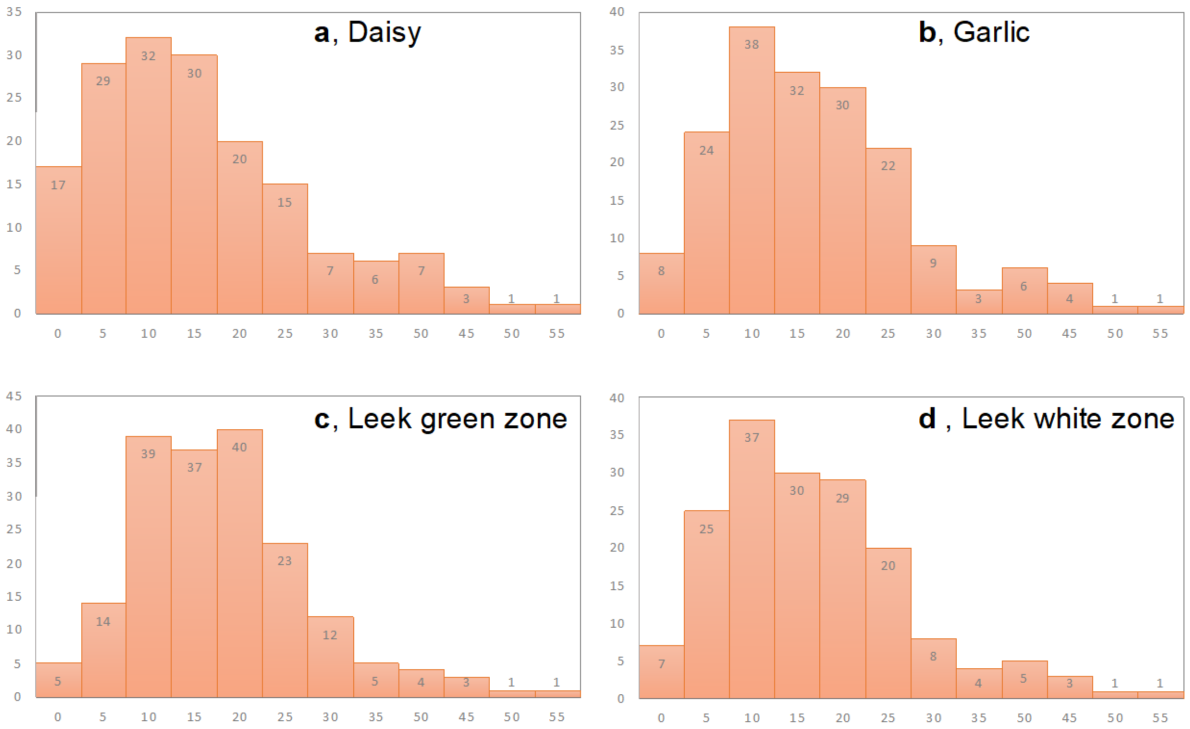

Figure S1. Statistical charts for AgNPs size.(a) Daisy; (b) Garlic; (c) Leek green zone; (d) Leek white zone

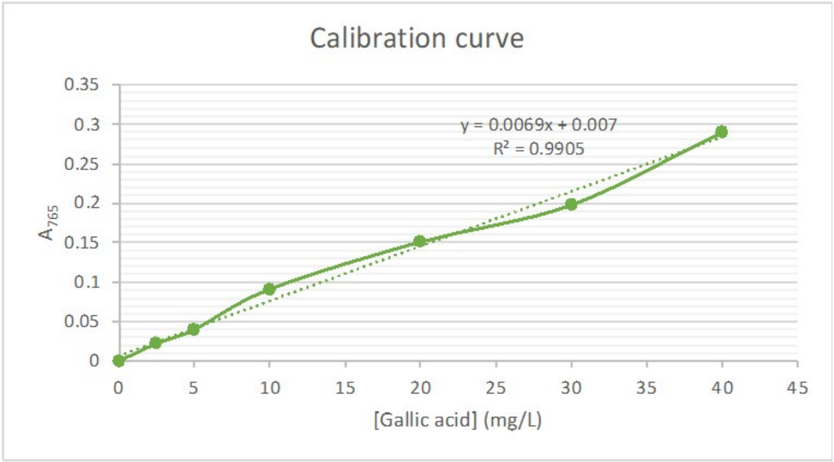

Figure S2. Calibration curve obtained with standard solutions of gallic acid.

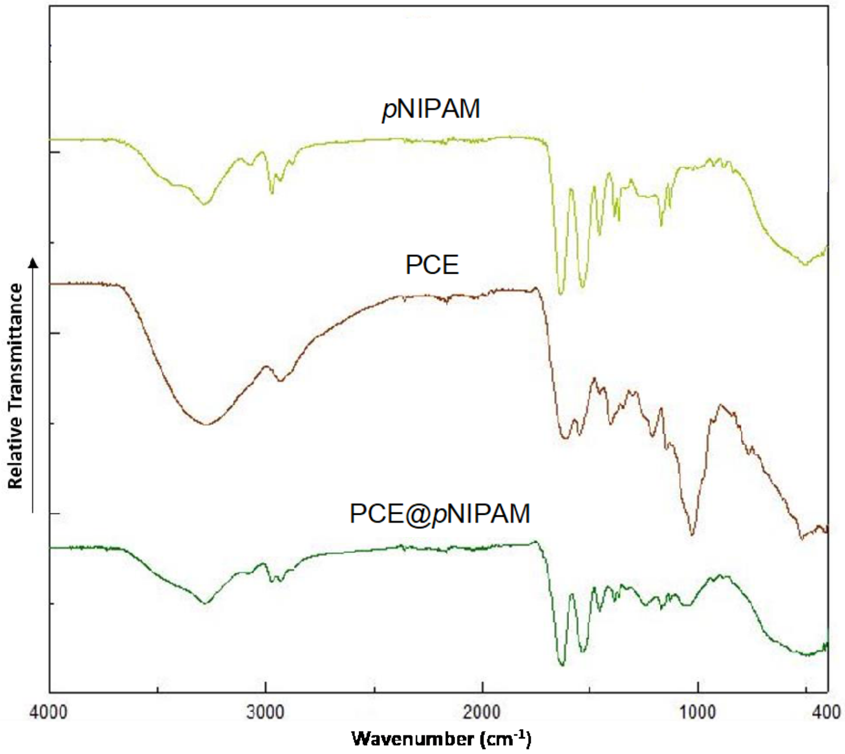

Figure S3. FT-IR Spectra of *p*NIPAM, PCE and PCE@*p*NIPAM.

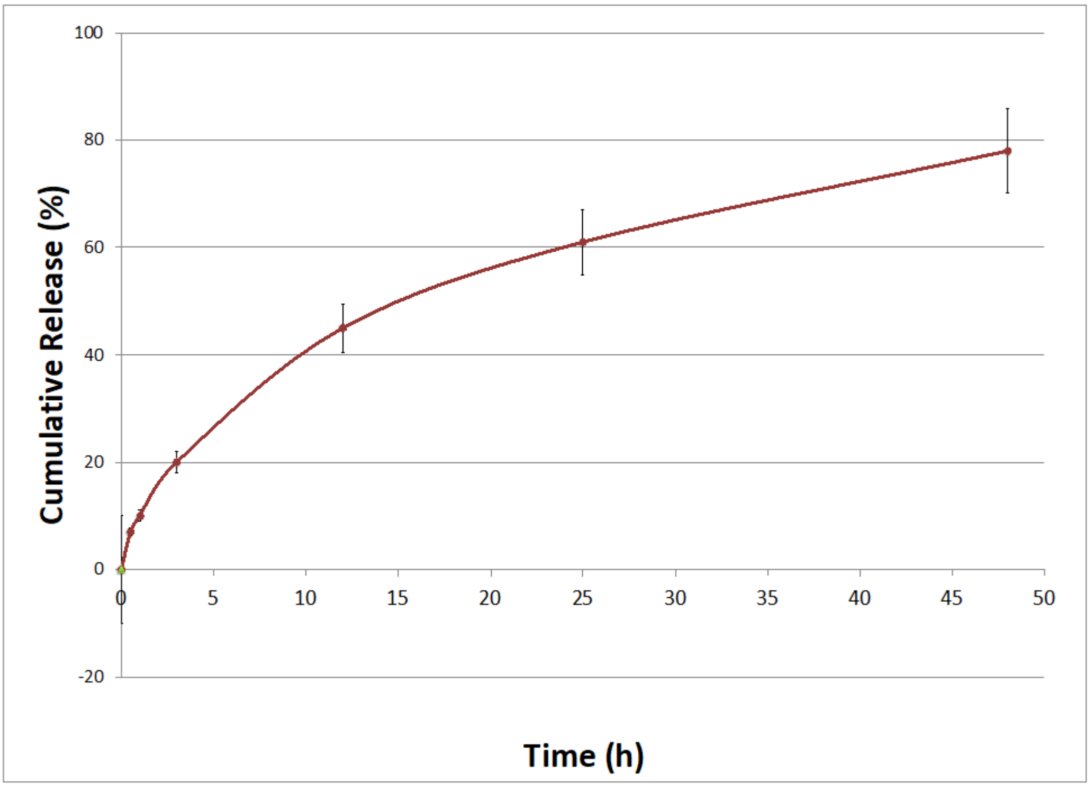

Figure S4. Cumulative MAAs release from PCE@*p*NIPAM.
